# Use of tracheobronchial tree 3-dimensional printed model: does it improve trainees’ understanding of segmentation anatomy? A prospective study

**DOI:** 10.1186/s41205-020-00092-3

**Published:** 2021-01-06

**Authors:** Christian O’Brien, Carolina A. Souza, Adnan Sheikh, Olivier Miguel, Timothy Wood

**Affiliations:** 1 University of Ottawa Faculty of Medicine, University of Ottawa, 451 Smyth Road Ottawa, Ontario, K1H 8M5 Canada; 2 Department of Medical Imaging, Ottawa Hospital Research Institute (OHRI), The Ottawa Hospital, University of Ottawa, 501 Smyth Road, Ottawa, K1H 8L6 Canada; 3 The Ottawa Hospital 3D Research Lab, 501 Smyth Road, Ottawa, K1H 8L6 Canada; 4 Department of Innovation in Medical Education (DIME), University of Ottawa Faculty of Medicine, University of Ottawa, 451 Smyth Road Ottawa, Ontario, K1H 8M5 Canada

**Keywords:** 3D printing, 3D-printed models, Computed tomography, Medical education

## Abstract

**Background:**

This prospective study investigated whether the use of 3D-printed model facilitates novice learning of radiology anatomy on multiplanar computed tomography (CT) when compared to traditional 2D-based learning tools. Specifically, whether the use of a 3D printed model improved interpretation of multiplanar CT tracheobronchial anatomy.

**Methods:**

Thirty-one medical students (10F, 21 M) from years one to three were recruited, matched for gender and level of training and randomized to 2D or 3D group. Students underwent 20-min self-study session using 2D-printed image or 3D-printed model of the tracheobronchial tree. Immediately after, students answered 10 multiple-choice questions (Test 1) to identify tracheobronchial tree branches on multiplanar CT images. Two weeks later, identical test (Test 2) was used to assess retention of information. Mean scores of 2D and 3D groups were calculated. Student’s *t* test was used to compare mean differences in tests scores and analysis of variance (ANOVA) was used to assess the interaction of gender, CT imaging plane and time on test scores between the two groups.

**Results:**

For test 1, 2D group had higher mean score than 3D group although not statistically significant (7.69 and 7.43, *p* = 0.39). Mean scores for Test 2 were significantly lower than for Test 1 (7 and 7.57, *p* = 0.03) with mean score decline for 2D group (Test 1 = 7.69, Test 2 = 6.63, *p* = 0.03), and similar score for 3D group (Test 1 and 2 = 7.43). There was no statistically significant interaction of gender and test score over time. Significant interaction between group and time of test was found for axial CT images but not for coronal images.

**Conclusions:**

Use of a 3D-printed model of the tracheobronchial anatomy had no immediate advantage over traditional 2D-printed images for learning CT anatomy. However, use of a 3D model improved students’ ability to retain learned information, irrespective of gender.

## Introduction

Knowledge of human anatomy is essential in Medicine and a crucial part of medical training. Traditionally, human anatomy teaching has been based on cadaveric-based training. Conventional 2-dimensional (2D) images, usually in the form of textbooks, remain the cornerstone of anatomy training after the initial cadaveric teaching [1]. Drawings of human anatomy are less costly and are widely available however they lack the three-dimensional orientation required for a deeper understanding of anatomic structures.

In recent years, 3-dimensional (3D) printing has emerged as a valuable resource for medical training, in addition to its utilization as a diagnostic and therapeutic tool. 3D printing uses digital data and specific software to create 3D objects by printing successive layers of material, such as plastic or metal [2]. As 3D printing has advanced and become more accessible, it has been increasingly utilized for anatomy training, being a cost-effective and more ethical alternative to cadaveric-based teaching [3–6]. Use of 3D printed models has the potential to provide trainees with better understanding of spatial anatomy thus potentially increasing their ability to orient themselves when assessing multiplanar 2D images. Previous research comparing the performance of 3D-printed models with other educational tools, such as cadaveric training and textbook images have shown positive results including a faster and more encompassing understanding of the human anatomy [2, 5, 6]. The advantages of 3D printing in medical education however may depend upon learners’ characteristics including spatial orientation skills and gender, among others. The effect of gender in spatial orientation tasks has been previously investigated with males typically outperforming females [7]. Interestingly, research has demonstrated that when using 3D printed models, most trainees focus mainly in the antero-posterior orientation, likely reflecting the better familiarity with coronal images commonly used in traditional learning materials such as textbooks [8, 9].

While 3D printed models have been proved valuable in human anatomy learning, it is not as clear whether 3D printing-based training facilitates learning of radiology anatomy. The interpretation of radiological images presents a unique challenge for trainees, as it requires visualization and interpretation of 3-dimensional anatomic structures from a 2-dimensional image. The goal of this study was to investigate whether the use of 3D printed model facilitates novice learning of radiology anatomy on multiplanar computed tomography (CT) when compared to traditional 2D-based learning tools. Specifically, we assessed whether the use of a 3D printed model improved interpretation tracheobronchial anatomy on multiplanar CT.

## Materials and methods

This prospective study was approved by the institutional research review board and the Vice-Dean of Undergraduate Medical Education. Medical students from years one to three were contacted by email sent on behalf of the principal investigator, the content of which was reviewed and approved by the Faculty of Medicine. For their participation in the research study, students were offered 1 h towards a medical education credit in anatomy and a certificate of participation provided by the Radiology Department. It was made clear that participation was voluntary and anonymous, that participants would be able to withdraw from the study at any point with no justification needed and would nonetheless be allowed to receive the aforementioned incentives.

A total of 31 medical students were recruited and matched for gender and level of training. Responders were randomly assigned to either the 2D or 3D group and all the subsequent responders were alternated between the two groups to ensure a similar number of participants in each group. Participants were assigned an identification number that was associated with their designation, gender, and level of training to ensure anonymization. The study occurred during September 1st, 2018 and January 31, 2019 from recruitment to completion of data collection.

**Fig. 1:**
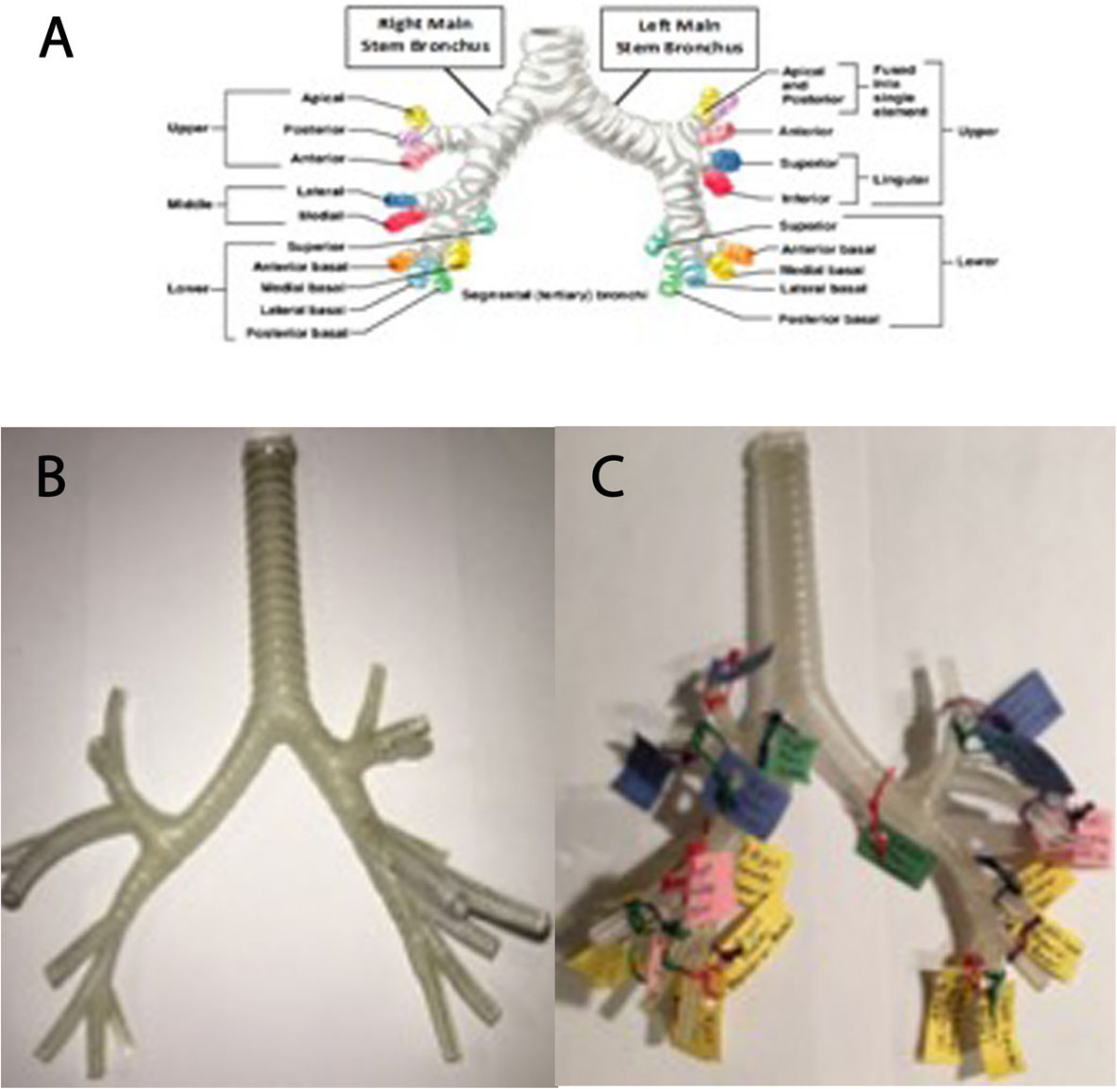
**a** 2D print of tracheobronchial tree used during learning activity. **b** frontal view of 3D-printed model used for the study. **c** same 3D printed model with labels matched with nomenclature provided in (**a)**

In the first phase of the study, students participated in a 20-min self-study session dedicated to learn the segmental anatomy of the tracheobronchial tree. Each 2D group student was provided with a 2D printed image of the tracheobronchial tree in anteroposterior projection (Fig. 1a) [10] whereas 3D group students were provided with a 3D printed model (Fig. 1b). The 3D printed model was designed with the 3-Matic Medical software (Materialise, Leuven, Belgium) based on CT images of the lung obtained with 1.5 mm thick slices. The CT images were chosen by a thoracic radiologist to ensure good anatomic representation and no congenital airway variants. The 3D model was printed with a Connex3 Objet500 3D printer using VeroClear material and printing time was 17 h and 47 min. The 2D and 3D materials had identical number of branches and identical nomenclature, as demonstrated on Fig. 1. In addition, the sizes of the models provided were approximately equal (8.5 × 11-in. page for the printed 2D and approximate similar size of the 3D model).

Upon completion of the allotted 20-min study period, the study tools were omitted and participants were asked to complete a multiple choice questions (MCQ) test in which they had to identify the correct bronchopulmonary segment on CT images. The test was created by an academic thoracic radiologist with over 15 years of experience, in consultation with a PhD in medical education, and reviewed by a 3rd year medical student to ensure quality of the images and appropriateness of the questions. The test consisted of 10 MCQ printed in a Word document. Each question showed a CT image in either coronal (three questions) or axial planes (seven questions) reconstructed with lung window settings. The branch to be named was identified with a red arrow. There were four choices (A to D) for each question containing universally accepted tracheobronchial tree nomenclature. The CT images used for the test were obtained from picture and archiving communicating system (PACS) from a single study. There were no anatomical variants in the case selected and all patient’s identifying information was removed. MCQ test was chosen because it most closely resembles the examinations used in an undergraduate medical education setting and was preferred over written answers because of the limitations to objectively quantify misspelled or partially correct answers in the latter.

Each student was assigned an identification number in the test sheet which was added to a separate and confidential spreadsheet with students’ demographics. The students had 10 min to complete the test. Participants’ scores were computed with one point given for each correct answer. Two weeks after the completion of Test 1, a follow-up test (Test 2) was conducted to assess learners’ retention of information. Participants were aware of the follow up test upon recruitment and knew that this was an optional component. The same MCQ test was sent by email as an attachment to all students involved. Participants were asked not to access study materials and to solely rely on information obtained from the initial study session. Students were given the same instructions as in Test 1. The scores of Test 2 were computed as per first test and added to the initial data.

The mean scores of the 2D and 3D groups for tests 1 and 2 were calculated and Student’s *t* test was used to compare mean differences in tests scores. Analysis of variance (ANOVA) was used to assess the interaction of gender, CT imaging plane and time (immediate, Test 1 and delayed, Test 2) on test scores between the two groups, calculated using SPSS Statistics. *p* < 0.05 was considered statistically significant.

## Results

The 31 students participating in Test 1 were comprised of 10 female participants, five randomized to the 2D and five to the 3D group. Of the 21 male students, 11 were randomized to the 2D and 10 to the 3D group. One female participant did not complete Test 2 and was excluded from the final analysis.

**Fig. 2:**
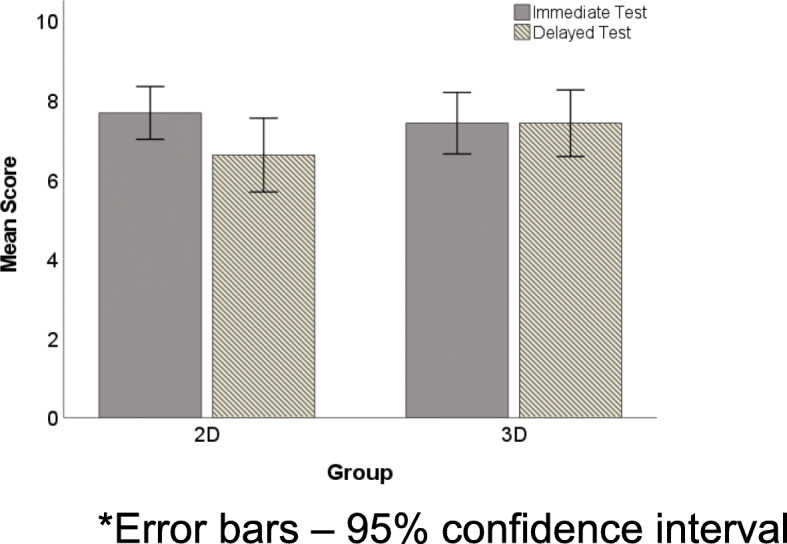
Bar plot displaying change in group score in Test 1 (immediate test) and Test 2 (delayed test). *Error bars – 95% confidence interval

Assessment of the effect of time on test scores between the two groups was tested using a 2 × 2 mixed ANOVA with group (2D and 3D) treated as a between-subjects variable, and time of the test (Test 1 and Test 2) treated as a within-subjects variable. The mean of the 2D group (7.69, SD 1.25) did not differ from the mean of the 3D group (7.43, F (1,28) = .32, *p* = 0.58, η_p_^2^ = 0.01) but there was a significant main effect of time (F (1,28) = 5.25, *p* = 0.03, and ɳ^2^_p_ = 0.16), with scores on Test 2 (7.00, SD 1.64) being lower than scores on Test 1 (7.57 SD 1.28) in the 2D group. More importantly, there was significant interaction between the test scores and time (F (1,28) = 5.25, *p* = 0.03, η_p_^2^ = 0.16). Table 1 displays the mean scores broken down by group (2D and 3D) and time (Test 1 and Test 2). As shown in Fig. 2, mean scores declined for the 2D group (Test 1 = 7.69, Test 2 = 6.63), but remained constant for the 3D group (Test 1 and Test 2 = 7.43).

**Table 1:** Effect of Time on Test Scores (2 × 2 mixed ANOVA) - mean test scores for 2D and 3D groups immediately after learning activity (Test 1) and 2 weeks later (Test 2)

		Test 1		Test 2	
Group	n	M*	SD	M*	SD
2D	16	7.69	1.25	6.63	1.45
3D	14	7.43	1.34	7.43	1.64
Total	30	7.57	1.28	7.00	1.64
Group – F(1,28) = .32, *p* = .58, ƞ_p_^2^ = .01					
Time – F(1,28) = 5.25, *p* = .03, ƞ_p_^2^ = .16					
Group x Time - – F(1,28) = 5.25, *p* = .03, ƞ_p_^2^ = .16					

*SD* standard deviation

*Score out of 10

Subsequent analyses were conducted to explore the role of gender and CT imaging plane in test scores. To assess the effect of gender and test scores for the two groups, a 2 × 2 × 2 mixed ANOVA was used, with gender and group treated as between-subjects variables, time treated as a within-subjects variable and overall test scores treated as dependent measure. There was no statistically significant interaction of gender and test score over time and in the two groups (F (1,26) = 0.98, *p* = 0.30), as demonstrated on Table 2. To investigate if CT imaging plane was an important predictor of test scores, separate analysis was performed using 2 × 2 mixed ANOVA with group (2D or 3D) as a between-subjects variable and time (Test 1 and Test 2) as a within-subjects variable conducted for axial and coronal CT planes. For axial CT images, a significant interaction between group and time (F (1,28) = 6.048, *p* = 0.02, ɳ^2^_p_ = 0.18) was found, and a non-significant main effect of group (F (1,28) = 0.435, *p* = 0.52, and ɳ^2^_p_ = 0.02) indicating that Test 2 scores were lower for the 2D group but not the 3D group. For coronal CT images, scores showed no significant difference between the groups, and no difference in groups with respect to time (Table 3).

**Table 2:** Effect of gender and time on test scores

Gender	Group	n	M	SD	M	SD
Male	2D	11	8.09	1.04	6.82	1.78
	3D	10	7.50	0.97	7.60	1.17
	total	21	7.81	1.03	7.149	1.54
Female	2D	5	6.80	1.30	6.20	1.79
	3D	4	7.25	2.22	7.00	2.16
	Total	9	7.00	1.66	6.56	1.88
Gender – F(1,26) = 1.71, *p* = .20, ƞ_p_^2^ = .06						
Group – F(1,26) = .47, *p* = .50, ƞ_p_^2^ = .02						
Gender x Group - F(1,26) = .25, *p* = .62, ƞ_p_^2^ = .01						
Time – F(1,26) = 3.86, *p* = .06, ƞ_p_^2^ = .13						
Time x Gender – F(1,26) = .10, *p* = .76, ƞ_p_^2^ = .00						
Time x Group – F(1,26) = 2.80, *p* = .11, ƞ_p_^2^ = .10						
Time x Gender x Group – F(1,26) = .49, *p* = .33, ƞ_p_^2^ = .04

**Table 3:** Effect of CT plane and time on test scores

			Immediate		Delayed	
View	Group	n	M	SD	M	SD
Axial	2D	16	4.75	1.13	3.63	1.75
	3D	14	4.50	1.34	4.50	1.45
	Total	30	4.63	1.22	4.03	1.65
Group – F(1,28) = .44, *p* = .52, ƞ_p_^2^ = .02						
Time – F(1,28) = 6.05, *p* = .02, ƞ_p_^2^ = .18						
Group x Time - F(1,28) = 6.05, *p* = .02, ƞ_p_^2^ = .18						
Coronal	2D	16	2.94	0.25	3.00	0.00
	3D	14	2.93	0.27	2.93	0.27
	Total	30	2.93	0.25	2.97	0.18
Group – F(1,28) = .51, *p* = .48, ƞ_p_^2^ = .02						
Time – F(1,28) = .28, *p* = .60, ƞ_p_^2^ = .01						
Group x Time - F(1,28) = .28, *p* = .60, ƞ_p_^2^ = .01						

## Discussion

The results of this study showed similar performance of students using 2D and 3D models when tested immediately after the learning activity. However, when assessing retention of knowledge, we found improved performance of those studying with a 3D printed model. The delayed test scores, 2 weeks after the learning activity, were lower for students using the 2D tool but remained constant for those using a 3D printed model. Previous studies have shown increased learner retention of information by way of visual learning methods when compared with conventional textbook or 2D-based learning [6, 11]. Our results suggest that this may be applied to learning of CT anatomy. In addition to retention of information, previous research has suggested that the use of visual models and more experiential learning can decrease the amount of time required to learn a particular topic or skill [2].

Our study also assessed whether gender influenced learning when using 2D or 3D models and whether performance differed for coronal or axial CT images. In regards to gender, previous research has suggested that males usually outperform females in spatial orientation tasks. The possible mechanisms and origin of such differences is outside the scope of this study and has been described elsewhere [7]. It is not clear however if this can be extrapolated to anatomy training using 3D printed models. Our results showed no statistically significant differences between female students’ scores in the 2D and 3D groups or when compared to male participants and improved retention was observed for both males and females in the 3D group. However, caution in interpreting this result is necessary due to the relatively small sample sizes of gender subgroups in our study. When assessing whether the use of a 2D or 3D tool influenced learning of CT images in different planes, we found that test scores for coronal CT images were similar for the 2D and 3D study groups and were very close to the 3.00 maximum score. Mean scores for axial CT images were also similar for the 2D and 3D groups at Test 1 but showed a larger decline for the 2D group at delayed testing (Test 2). The better performance in the interpretation of coronal CT images and the improved retention of knowledge may reflect the greater familiarity of novice learners with chest anatomy in the antero-posterior (coronal) orientation such as provided by many anatomy imaging resources and wide exposure to chest radiographs. While radiologists are trained to review chest CT images primarily in the axial planes, trainees are less familiar with the anatomy and spatial orientation of axial images. It has been previously reported that when learning with 3D printed models, trainees tend to use predominantly the anterior-posterior view [8]. In our study however, the better performance with coronal CT images as compared to axial images should be interpreted with caution as the small number of questions (three) for each CT plane may have hampered accurate interpretation of results. Nonetheless, the preference to utilize 3D printed model in the anteroposterior view may decrease the inherent advantages of a spatial model to learn multiplanar anatomy. As 3D printed models are increasingly used in medical education, teachers and trainees must be encouraged to explore the full potential of these tools.

Our study has a few limitations. First, in spite of the prospective design, due to the relative small number of participants some of the results need to be interpreted with caution, notably the effect of gender and CT plane tested. Secondly, a confounding factor that was unable to be circumvented was that all students had been previously exposed to respiratory system anatomy. Nonetheless, considering the novice level of training, exposure to CT anatomy of the tracheobronchial tree was somewhat limited. One potential issue to be mentioned is that many students had never used a 3D printed model for anatomy learning before the study. First time use of a more complex model may be overwhelming and utilization may have been suboptimal. Assessment of the potential learning curve of 3D model-based learning would require a longitudinal study of 3D use over time compared to 2D traditional learning.

## Conclusion

In summary, our study showed that the use of a 3D printed model of the tracheobronchial anatomy had no immediate advantages over traditional 2D textbook images for learning of CT anatomy. However, use of a 3D printed model increased the students’ ability to retain the learned information, irrespective of gender.

## Data Availability

The data collected in the study is maintained in password-protected cloud-based files and are available from the corresponding author on reasonable request. Hardcopy material (exams performed by participants) is kept in a locked cabinet in the office of research of the Department of Radiology at the University of Ottawa, The Ottawa Hospital.

## Abbreviations

2D: Two dimensional
3D: 3-dimensional
CT: Computed tomography
MCQ: Multiple-choice questions
PACS: Picture and archiving communicating system
ANOVA: Analysis of variance
SD: Standard deviation
